# Cognitive Behavioral Treatment to Improve Adherence to Hemodialysis Fluid Restrictions: A Case Report

**DOI:** 10.1155/2009/835262

**Published:** 2009-12-28

**Authors:** Heather M. Anson, Michelle R. Byrd, Ellen I. Koch

**Affiliations:** ^1^Psychology Department, Eastern Michigan University, Ypsilanti, MI 48197, USA; ^2^Naval Medical Center San Diego, San Diego, CA 92108, USA

## Abstract

This case report describes outpatient psychological treatment targeting adherence to fluid restrictions in a hemodialysis patient. The consequences of nonadherence to fluid restrictions in hemodialysis patients range from minor discomfort to increased hospitalizations and mortality rates. In addition, when patients chronically fail to adhere, they may no longer be candidates for kidney transplant. The interventions focused on polydipsia, characterized by excessive fluid intake. The methods involved 11-sessions of individual psychotherapy incorporating strategies including increasing awareness, decreasing motivation, increasing effort, engaging in competing events, conducting thought stopping, breaking repetitive routines, eliciting social support, and receiving reinforcement. Results demonstrated that the patient successfully restricted his fluid intake at or below recommended levels 83% of days after fading of treatment began. This case report demonstrates the success of cognitive behavioral treatment strategies with a nonpsychiatric hemodialysis patient.

## 1. Introduction

In the recent years, patients with end-stage renal disease (ESRD) have increased life expectancies due to advances in hemodialysis.Hemodialysis as used in the case of kidney failure involves dialysis of the blood to remove toxic substances or metabolic wastes from the bloodstream. Such procedures serve as a bridge to kidney transplant or a means to manage ESRD in a way that increases both the length and quality of life for patients who are not candidates for transplant.

Hemodialysis involves a complex treatment regimen, and to be successful requires a number of self-management tasks, including maintenance of a polypharmacological treatment regimen, careful monitoring and control over dietary and fluid intake, and scheduling, attending, and completing the dialysis sessions themselves (which may total 9–15 hours/week). It has been estimated that more than 50% of hemodialysis patients are nonadherent in some manner within a one-month time period [1] and upwards of 70% of patients fail to adequately restrict their fluid intake [2]. These rates are alarming, particularly with the consideration that even occasional episodes of serious noncompliance can have severe adverse effects. For example, the consequences of failing to adequately restrict fluids range from increased discomfort and side effects during and immediately after dialysis sessions (e.g., cramping, emesis, syncope) to congestive heart failure, increased hospitalizations, and death when patients are chronically nonadherent [3].

Several attempts have been made to improve patient adherence to hemodialysis treatment recommendations regarding fluid restrictions. The most rigorous study to date conducted a randomized controlled trial of group cognitive behavioral therapy to improve adherence to fluid restrictions [4]. Although no improvements in fluid intake were found during the 4-week treatment phase, there were significant differences (*P* < .001) at 10-week followup, suggesting that treatment improved adherence over time. Similarly, Hegel et al. [5] compared cognitive and behavioral strategies to improve adherence to fluid restrictions in a small sample (*n* = 4) of male hemodialysis patients. Both interventions were equally successful during the treatment phase, but the effects of behavioral strategies resulted in maintenance of adherent behavior at a two-month followup. Furthermore, rates of adherence did not improve with a combination of cognitive and behavioral interventions. Unfortunately, the data from these studies are not yet sufficient to suggest a standard of practice in improving adherence to fluid restrictions in hemodialysis patients.

In the current case report, cognitive behavioral strategies were used to improve fluid adherence in a hemodialysis patient. The main treatment goal for the patient involved decreasing a behavior, namely, fluid intake. The current patient was unwilling to engage in emotionally focused psychotherapy, and he specifically requested behavioral treatment. Therefore, the researchers chose to use interventions consistent with habit reversal strategies. There are many similarities between habit reversal strategies [6] and typical psychological treatment for hemodialysis patients. The main difference in habit reversal is the lack of exploration of underlying cognitive and emotional processes. Treatment components emphasizing habit reversal strategies included increasing awareness, decreasing motivation, increasing effort, engaging in competing events, conducting thought stopping, breaking repetitive routines, eliciting social support, and receiving reinforcement.

## 2. Case Report

### 2.1. Patient History

The patient was a 60-year-old, married, Caucasian male. He was a highly educated college professor in a health related field. The patient had been diagnosed with type II diabetes, ESRD, and congestive heart failure (CHF). The patient had been dependent on three weekly hemodialysis sessions for four years. He was a candidate for kidney transplant, but his status was deactivated due to chronic nonadherence to fluid restrictions resulting in fluid overload. He presented for cognitive behavioral treatment due to difficulties tolerating the fluid restrictions set forth by his renal specialist.

The patient's doctors recommended that he consume less than 48 to 60 ounces of fluid per day. Upon entering cognitive behavioral treatment, the patient reported fluid consumption of approximately 90 to 150 ounces per day, which was 2 to 4 times greater than the recommended amount. Ninety percent of these liquids included caffeinated sodas and water. The other ten percent included foods such as soup and gelatin.

The patient reported that he typically consumed fluid excessively during repetitive tasks such as grading papers, lecturing, driving, or watching television. Prior to fluid consumption, he stated that he generally felt an urge to “quench his thirst” due to sensations of a dry throat or mouth. In addition, he also engaged in fluid consumption “out of habit,” as he had arranged his environment in such a way that fluid intake required little response effort (e.g., small refrigerator in his office, television trays with cups and bottles in his leisure areas).

### 2.2. Cognitive Behavioral Treatment Strategies

As previously noted, treatment consisted of eight main components to focus on decreasing the patient's excessive consumption of liquid. A detailed description of treatment sessions is available from the first author.

#### 2.2.1. Awareness Training

Awareness training was introduced during session 1 and adjusted as necessary throughout subsequent sessions. The purpose of awareness training was to assist the patient in becoming attentive to his fluid intake behavior including associated stimuli, environments, and thoughts [7]. Initially, the therapist provided the patient with self-monitoring forms to track daily fluid intake. As the patient was resistant in completing the record forms each day, he agreed to telephone in his fluid intake daily. He phoned in his fluid intake 93% of the 112 treatment days, making him highly compliant with this treatment strategy. He reported that phoning in consumption of fluids was helpful, as this approach made him feel accountable on a daily basis. As the patient's fluid intake began to stabilize, phone reports were faded to three times a week, then to one time per week. 

In addition to daily fluid monitoring, the patient completed thought records associated with urges to consume fluid. These thought records emphasized rational emotive behavior therapy approaches [8, 9]. The patient had difficulty completing thought records on a daily basis, but reported that when completed, they stopped him from excessively consuming fluids.

Finally, a fluid intake schedule was created in an effort to weaken the association between thirst and fluid consumption. The patient and therapist created a daily fluid intake schedule based on the patient's monitoring of fluid consumption throughout his day. This schedule involved having the patient consume predetermined amounts of fluid at scheduled intervals throughout the day, rather than in response to internal (e.g., thirst, boredom) or external (e.g., seeing a cup) antecedents. The patient reported the schedule to be successful in keeping him alert to his fluid consumption and helped him “stay on track” throughout the day.

#### 2.2.2. Decrease Motivation

Decreasing motivation was introduced during session 2. Caffeinated soda composed a large volume of the patient's consumed liquids. The patient was asked to eliminate caffeine from his diet due to the dehydrating effects of caffeine [10]. Once the patient eliminated caffeinated sodas from his home, thereby also eliminating a cue to consume fluid, he was successful in decreasing caffeine intake. He replaced some of his caffeinated soda with decaffeinated soda, but by the end of treatment, approximately 90 percent of the patient's fluid intake consisted of water.

#### 2.2.3. Increase Effort

Increasing effort [11] was introduced during session 2 and adjusted as necessary throughout subsequent sessions. The patient consumed most of his liquids during repetitive activities such as watching television, completing office work, giving classroom lectures, and driving. In order to increase the effort required to consume fluid, the patient removed all cups and bottles from his work and leisure areas. To eliminate fluid intake in the patient's car, he stopped buying drinks from fast food restaurants. In addition, it was recommended that he limit his fluid intake to eating areas such as the kitchen or cafeteria to gain stimulus control over his fluid consumption.

Even with drinking containers removed from leisure areas, the patient continued to go to the refrigerator in his home for drinks often. To assist in decreasing trips to the refrigerator, the patient self initiated “barricading” the refrigerator with chairs and used visualizations such as warning/danger signs.

#### 2.2.4. Competing Events

Competing events were introduced during session 2 and adjusted as necessary throughout subsequent sessions. Competing events involve patients engaging in responses that are mutually exclusive from the behavior excess [12]. The patient and therapist devised a list of competing events to engage in when the urge to consume fluid arose. Some examples of competing events included relaxation (deep breathing), slow exercises, playing with his dog, hugging his wife, and telling his wife he loved her. The patient reported these activities to be helpful in decreasing his excessive fluid intake.

#### 2.2.5. Thought Stopping

Thought stopping was introduced during session 7. Thought stopping is a method used to interrupt undesirable or unproductive thoughts [13]. The therapist and patient developed a “thought-stop” card of phrases based on the thought record that were incompatible with urges to consume fluid. Examples of phrases included “Overdrinking makes me feel bloated” and “I have control over my urges, they do not control me.” The patient read this card when the urge to consume fluid arose as well as several times throughout the day to internalize work done with the thought records.

#### 2.2.6. Break Repetitive Routines

Breaking repetitive routines, or competing response training [14] and precomittment [15], was introduced during session 2. In order to break up time-periods where the patient was most likely to engage in excessive fluid consumption (e.g., during office work or when watching television), the patient set a timer. Initially the patient set the timer for 10 minutes. Each time the timer rang the patient got up to stretch, or walk around. As the patient's fluid intake behavior began to stabilize, the timer was faded to 20 minutes, and then to more natural occurring breaking points (e.g., completion of a specific amount of office work, television commercials, etc.)

#### 2.2.7. Eliciting Social Support

Eliciting social support [16] was introduced during session 2. The patient elicited social support from the people in his life to assist in managing his fluid intake. He shared his excessive fluid consumption struggles with his wife and students and asked them to help keep him accountable. This included others reminding him not to consume fluid in unauthorized areas, socially reinforcing him for not consuming fluids excessively, and pointing out when he was not following his other treatment strategies. The patient reported that this social support component was helpful in keeping him accountable and motivated. It was also helpful in avoiding relapse, as he did not like the idea of others seeing him fail.

#### 2.2.8. Reinforcement

Reinforcement was introduced during session 2. The patient was asked to reward himself with pleasurable activities at the end of days he was able to successfully keep his fluid intake under the recommended amount [17]. The most motivating reinforcing activity for the patient was taking his wife out for the evening. However, he did not engage in these types of reinforcing activities each day he consumed less than 60 ounces of fluid due to factors such as fatigue on dialysis days. The patient reported that this intervention was reinforcing and helped motivate him to manage his level of fluid intake throughout the day.

### 2.3. Outcomes

**Figure 1:**
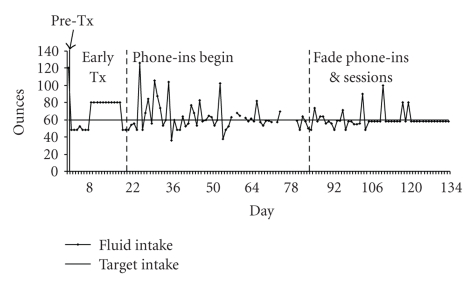
Patient's daily fluid intake levels from pretreatment to termination. Note: breaks in the graph indicate days when fluid intake level was not recorded.

The patient appeared to be motivated and committed to the cognitive behavioral intervention. He readily engaged in assessment, intervention, and evaluation with the therapist during each session and expressed pride in his improvement over the course of the intervention. Initial self-report indicated that the patient was consuming 90 to 150 ounces of fluid each day. According to his subsequent self-monitoring reports and as illustrated in Figure 1, he was able to decrease his fluid intake to less than 60 ounces per day by the conclusion of treatment. In addition, throughout the course of treatment, a downward trend of relapse frequency and total amount of liquids consumed on those relapse days decreased.

## 3. Discussion

The current case report illustrates the successful use of cognitive behavioral therapy intervention strategies with a patient diagnosed with end-stage renal disease who presented for treatment due to difficulties tolerating fluid restrictions set forth by his renal specialist. The treatment was implemented successfully in an outpatient setting with 1150-minute sessions. This case study clearly demonstrates the successful application of a combination of well researched interventions [6, 13, 18] in an uncontrolled, clinical setting.

In the previous studies researchers have attempted to identify underlying cognitive and emotional processes to investigate factors associated with nonadherence to medical regimens such as perceived locus of control [19] and ways of coping with illness-related stress [20, 21]. The present case study did not focus on underlying emotional or dynamic factors as etiology; instead focus was placed solely on increasing adherence. To achieve adherence, the symptom of polydypsia was targeted immediately and directly with no apparent adverse affect on treatment gains. As demonstrated in this case report, focus primarily on fluid adherence may allow for a simplified and possibly briefer treatment protocol, particularly when the patient's health may be further threatened by a delay in treatment gains. However, the importance of factors that play a role in the etiology, progression, and maintenance of nonadherence should not be minimized. 

The patient in this case study demonstrated reduction in fluid intake early in treatment. This is in contrast to Sharp et al. [4] where no improvements in fluid intake were seen until after a 10-week treatment followup and consistent with the findings of Hegel et al. [5]. Possible differences giving rise to more immediate gains in the current case may have been the combination of cognitive and behavioral treatments in an individual rather than group format. Of note, the patient in this case report responded positively to the daily accountability of phoning in his fluid intake, which is a novel form of self-monitoring. Requiring accountability in intervals between therapy sessions may be an important component in fluid adherence, but requires future research.

The patient in this case report also responded positively to the incorporation of cognitive and behavioral strategies, which is similar to Hegel et al.'s [5] finding that behavioral and cognitive therapies were equally successful during the treatment phase. However, upon a two-month followup these researchers found behavioral strategies to be the factors maintaining adherent behavior [5]. Because the present patient received both types of intervention, it is not known what particular techniques, if any, will allow him to maintain gains. This is a limitation of the study. The methodology used does not allow one to determine, with any degree of confidence, the variable or variables responsible for the reported fluid intake reductions. Furthermore, the experimental design does not allow one to determine if some treatment components were more effective than others. It is possible that only one of the interventions, such as awareness training, would have been necessary to produce the reported decrease in fluid intake. This lack of experimental control precludes the researchers from demonstrating a functional relationship between the intervention components and fluid intake. In order to demonstrate a functional relationship additional research is required to specifically show that as the intervention varies, the amount of fluid intake likewise varies. It would be possible to accomplish this task using single subject designs such as a changing criterion design [22], or multiple baseline across participants design [23]. Without this demonstration of experimental control, it is possible that variables other than those in the treatment package (e.g., talking to the patient's doctor or wife) are responsible for the patient's dramatic fluid decrease. In addition, it should be noted that this patient is an individual with many resources (e.g., financial, strong social support) who is intellectually functioning at an above average level. These factors likely impact his responsiveness to treatment and overall motivation toward improved adherence. Thus, the findings from this case report may have limited generalizability to the ESRD population.

Another weakness of the current case report is the absence of followup data following termination of treatment. Additional studies are required to determine the most effective treatment strategies in assisting dialysis patients in adherence to fluid restrictions as well as the best strategies to maintain their gains. A final weakness of the current report is the lack of interdialytic weight gain (IWG) measures, which is the accepted manner of evaluating adherence to fluid restrictions. There is an inherent danger in relying solely on patient report of consumed liquid as we did in this case due to the possibility of biased or otherwise erroneous reporting. However daily self-report, as opposed to typical weekly self-report, increases reporting accuracy [24–26]. It will be important for future researchers to investigate how self-report of fluid intake behavior and IWG compare. In addition, treatment integrity checks should be incorporated into future investigations.

The current case report highlights the efficacy of combining cognitive and behavioral interventions. After cognitive and behavioral treatment, the patient's eligibility to be considered for a kidney transplant was reactivated, as he successfully demonstrated control over his fluid intake. This case demonstrates the utility and efficacy of applying cognitive behavioral techniques to improve response to medical interventions.
